# Enthalpy-entropy compensation at play in human copper ion transfer

**DOI:** 10.1038/srep10518

**Published:** 2015-05-27

**Authors:** Moritz S. Niemiec, Artur P. G. Dingeldein, Pernilla Wittung-Stafshede

**Affiliations:** 1 Department of Chemistry, Umeå University, 901 87 Umeå, Sweden

## Abstract

Copper (Cu) is an essential trace element but toxic in free form. After cell uptake, Cu is transferred, via direct protein-protein interactions, from the chaperone Atox1 to the Wilson disease protein (WD) for incorporation into Cu-dependent enzymes. Cu binds to a conserved C_1_XXC_2_ motif in the chaperone as well as in each of the cytoplasmic metal-binding domains of WD. Here, we dissect mechanism and thermodynamics of Cu transfer from Atox1 to the fourth metal binding domain of WD. Using chromatography and calorimetry together with single Cys-to-Ala variants, we demonstrate that Cu-dependent protein heterocomplexes require the presence of C_1_ but not C_2_. Comparison of thermodynamic parameters for mutant versus wild type reactions reveals that the wild type reaction involves strong entropy-enthalpy compensation. This property is explained by a dynamic inter-conversion of Cu-Cys coordinations in the wild type ensemble and may provide functional advantage by protecting against Cu mis-ligation and bypassing enthalpic traps.

Transient protein-protein and protein-ligand interactions are fundamental components of biological activity. To understand such reactions, not only are the structures of the involved proteins important but also the energetics of the individual steps. Formation of protein-protein and protein-ligand complexes is often favored thermodynamically due to an increase in negative enthalpy or positive entropy, or a combination of both. In many signaling and transport pathways in living systems, transient protein-protein and protein-ligand complexes result in directional transfer of a ligand or signal. In such cases it may not be trivial to deduce the energetic components that are involved in every step of the path as pure intermediates are hard to isolate and reactants as well as products often have similar properties. In human Cu transport, Cu is shuttled from one protein to another to eventually become loaded on Cu-dependent enzymes^1^,^2^. Despite structural work on the proteins involved in this metal transport chain, the molecular mechanisms and driving forces are not understood. In part, this knowledge gap is due to the fact that the involved proteins have similar folds and Cu-binding sites.

Cu is found in the active sites of proteins that participate in cellular reactions such as respiration, antioxidant defense, neurotransmitter biosynthesis, connective-tissue biosynthesis and pigment formation^3^,^4^,^5^. The ability of Cu to oxidize/reduce (switch between Cu^+^ and Cu^2+^) allows Cu-containing proteins to play important roles as electron carriers and redox catalysts in living systems. To avoid toxicity of Cu^+^, the intracellular concentration of Cu is regulated *via* dedicated proteins that facilitate its uptake, efflux as well as distribution to target Cu-dependent proteins and enzymes^1^,^2^,^6^. In humans, the 68-residue Cu^+^ chaperone Atox1 picks up Cu^+^ that has entered the cell via CTR1 and delivers the metal to cytoplasmic metal-binding domains in ATP7A and ATP7B (also called Menke’s and Wilson disease proteins, respectively), two homologous multidomain P_1B_-type ATPases located in the trans-Golgi network. Many human Cu-dependent enzymes acquire Cu^+^ in the Golgi before reaching their final destination^1^,^2^,^6^. In both Menke’s and Wilson disease proteins (MK and WD, respectively), there are six metal-binding domains in the N-terminal cytoplasmic part^7^. In contrast to humans, bacterial and yeast P_1B_ type ATPases have only one or two metal-binding domains and the purpose for as many as six metal-binding domains in WD and MK has been proposed to be regulation of activity. During the catalytic cycle, which ultimately results in Cu^+^ transfer to the opposite side of the membrane, WD and MK are likely to undergo significant conformational changes^8^. An interaction between the ATP-binding domain and a construct of the six metal-binding domains of WD was reported^9^; this interaction was found to depend on the metal-loading status as well as on phosphorylation events^10^. In further support of regulatory roles for the metal binding domains, the corresponding Cu^+^ chaperone in bacteria was found to bypass the metal-binding domain of the bacterial P_1B_-type ATPase and instead deliver Cu^+^ directly to the ATPase membrane entry site^11^. Moreover, based on the crystal structure of a bacterial homolog^12^, a putative docking site for the chaperone at the membrane entry site was identified. Nonetheless, some studies have shown that the presence of the human and yeast metal-binding domains, at least some of them, is essential for Cu^+^ transfer activity^13^,^14^.

Cu^+^ chaperones and target domains from many different organisms possess the same ferredoxin-like fold and Cu^+^-binding motif^15^. The six domains, as well as Atox1, bind Cu^+^ via two conserved Cys residues in a surface-exposed MTC_1_XXC_2_ (X, any residue) Cu-binding motif^3^,^15^. Earlier *in vitro*^16^,^17^,^18^,^19^,^20^,^21^ and *in silico*^22^ work has shown that Cu^+^ transfer from Atox1 to metal-binding domains of WD and MK proceeds in a two-step process, via Cu-bridged heterodimeric protein complexes (Step 1: formation of an Atox1-Cu-WD complex; Step 2: decomposition of protein heterocomplex into products; Fig. 1) where the metal is shared between the two metal-binding sites.

Cu^+^ is thought to move from one protein to the other via exchange reactions involving Cu^+^-sulfur intermediates^19^. All six domains of WD and MK can be metalated by Cu-Atox1 but only in some cases, have Cu^+^-dependent complexes been detected by NMR via slowed tumbling times^16^,^17^,^23^,^24^. Based on affinity and NMR studies, Cu^+^ binding to a WD domain is favored over binding to Atox1 by a factor of 3–5. We showed earlier that upon mixing of Cu^+^-Atox1 and the fourth metal-binding domain of WD (WD4), a stable ternary complex is formed in equilibrium with both substrates and products. In contrast, when the interaction between a two-domain construct of domains 5 and 6 in WD and Cu^+^-Atox1 was investigated, the protein-protein interaction did not survive gel filtration but still allowed for Cu^+^ transfer^25^. For Atox1 and WD4, we used size exclusion chromatography (SEC) in combination with titration calorimetry to analyze the reaction in Fig. 1^26^ and we found that Atox1-Cu-WD4 protein heterocomplex formation is driven by favorable enthalpy and entropy changes, whereas the overall reaction, from Atox1 to WD4, relies on only enthalpy.

Here we dissect the Cu^+^ transport reaction on a molecular level by identifying what Cu-sulfur coordinations are populated in the ensemble of Atox1-Cu-WD4 heterocomplexes, along with a mechanistic explanation based on thermodynamics. We show that Cu^+^-dependent protein heterocomplexes only form when the 1^st^ Cys in the metal-binding motif of either protein is intact. Thermodynamic analysis of mutant Atox1-Cu-WD4 heterocomplexes points to enthalpy-entropy compensation in the wild type system: this is explained by dynamic interconversion of the two identified tricoordinate Cu^+^-Cys sites in the wild type protein heterocomplex.

## Results

**Figure 1:**
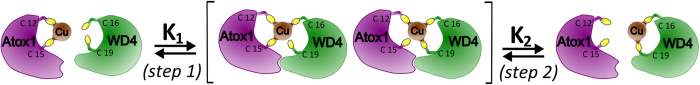
Illustration of 2-step Cu transfer from Atox1 to WD4 showing two intermediate protein heterocomplexes with different Cu-cysteine coordinations. Out of four possible tri-coordinate Cu^+^-cysteine complexes, only two (the ones shown) are observed *in vitro*. Purple: Atox1; green: WD4; yellow: Cys residues in metal-binding motifs.

Previous NMR findings for the yeast proteins Atx1 and Ccc2, and the human Atox1 and MK1 pair, have suggested that C_1_ in the metal-binding motif in each protein is essential for protein hetercomplex formation in the presence of Cu^+^^21^,^27^. To address this for the Atox1-WD4 system (Fig. 1), and to characterize the underlying thermodynamic forces, we here used a different, more direct, approach in which a combination of SEC, calorimetry and protein engineering was applied. The SEC approach has been benchmarked for the wild type protein system^26^ and is based on the fact that (1) WD4 elutes at a different position as compared to Atox1 and (2) changes in 254/280 nm absorption ratio reports on Cu^+^ loading status of individual proteins (Fig. S1A-D). When wt Atox1 is mixed with wt WD4 and one protein is in its holo form, the resulting elution profile then reveals a mixture of five species as shown in Fig. 1: apo and holo Atox1, apo and holo WD4 and an ensemble of Cu^+^-bridged protein heterocomplexes^26^ (Fig. S1F-H).

**Figure 2:**
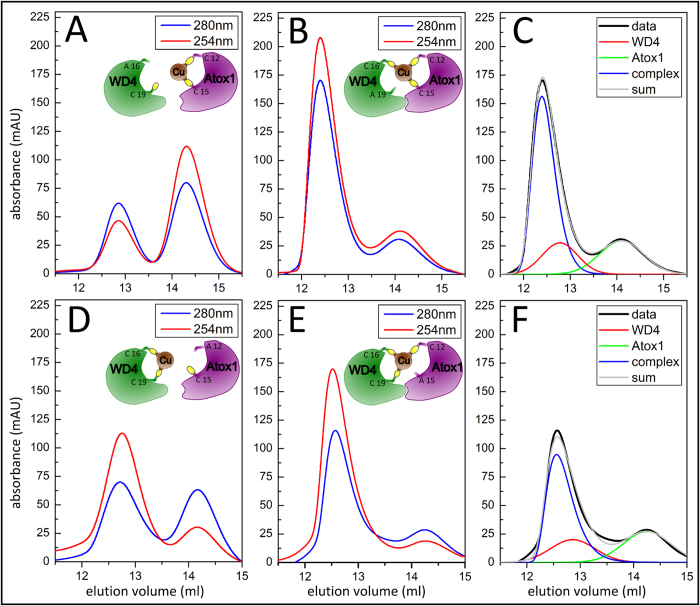
Assessment of protein heterocomplex formation with mutant proteins. SEC analysis probed by dual wavelength (254 and 280 nm) absorption. (**A**) WD4_A1C2_ + Cu^+^-Atox1: (**B**) WD4_C1A2_ + Cu^+^-Atox1, (**D**) Cu^+^-WD4 + Atox1_A1C2_, and (**E**) Cu^+^-WD4 + Atox1_C1A2_. As depicted in the illustrations in the insets, there is no interaction for **A** and **D**, but protein heterocomplexes form in **B** and **E** mixtures. (**C**) and (**F**): Deconvolution of the 280 nm traces in **B** and **E** into 3 components using fityk®: experimental trace data (black; named ‘data’), as well as deconvoluted traces for individual WD4 (red), individual Atox1 (green), the protein heterocomplex (blue), and the sum of the three deconvoluted signals (grey).

We here prepared four single Cys-to-Ala variants: Atox1_C1A2_, Atox1_A1C2_, WD4_C1A2_, and WD4_A1C2_. The individual point mutated variants are all folded as indicated by single SEC elution peaks at the same position in the chromatogram as the corresponding wt protein (Fig. S2). As expected since both Cys sulfurs are needed in order to bind Cu^+^ to individual proteins, upon addition of Cu^+^, the 254/280 nm absorption ratio remains that of the apo forms. To probe Cu^+^ dependent protein-protein interactions, we performed a set of SEC experiments with the four mutant proteins individually mixed with the partner protein in its wild type Cu^+^-loaded form. As visualized in Fig. 2, we find that when C_1_ in each protein is mutated to Ala, no heterocomplex is formed and the wt protein loaded with Cu^+^ prior to mixing remains in its holo form (Fig. 2A,D). In contrast, when C_2_ in each protein is mutated to Ala and mixed with the wild type Cu^+^-loaded partner, after SEC some protein is lost from the monomeric protein fractions and a new peak arises demonstrating the presence of a protein heterocomplex (Fig. 2B,C,E,F). Taken together, of the four possible combinations of three Cys residues as Cu ligands, only two (Atox1_C1C2_ and WD4_C1_; Atox1_C1_ and WD4_C1C2_) assemble into Cu^+^-dependent protein heterocomplexes.

From the SEC profiles of the two productive Atox1-WD4 mutant mixtures we determined the concentration of free Atox1 from the 280 nm absorption and, via comparison to the expected concentration from elution of an individual protein sample, we determined how much protein is lost from the monomeric peak and instead engaged in protein heterocomplexes. Assuming 1:1:1 protein heterocomplexes^21^,^26^,^27^, we can then calculate the concentration of protein heterocomplex, and therefore also how much monomeric WD4 is left (since the starting concentrations of both proteins are known). From these concentrations, apparent K_D_ values for the two mutant protein heterocomplexes Atox1_C1C2_-Cu-WD4_C1A2_ and Atox1_C1A2_-Cu-WD4_C1C2_ are determined (Table 1). Importantly, since the Atox1 peak alone can be used to derive all three concentrations involved in each of the two equilibria probed, any partial dissociation of the protein heterocomplex upon dilution on the column will not affect the K_D_ estimates. To emphasize that the SEC approach is not a true equilibrium method, we term the derived K_D_ values as apparent. Formation of the Atox1_C1C2_-Cu-WD4_C1A2_ complex reports on Step 1 in Fig. 1 starting from the left, whereas formation of the Atox1_C1A2_-Cu-WD4_C1C2_ complex reports on the reverse of Step 2 in Fig. 1, i.e., starting from the right. (We note that the absorption at 280 nm of the heterocomplex elution peak cannot be used to derive protein heterocomplex concentration since the extinction coefficient at 280 nm changes when the proteins engage in this complex^26^.)

**Figure 3:**
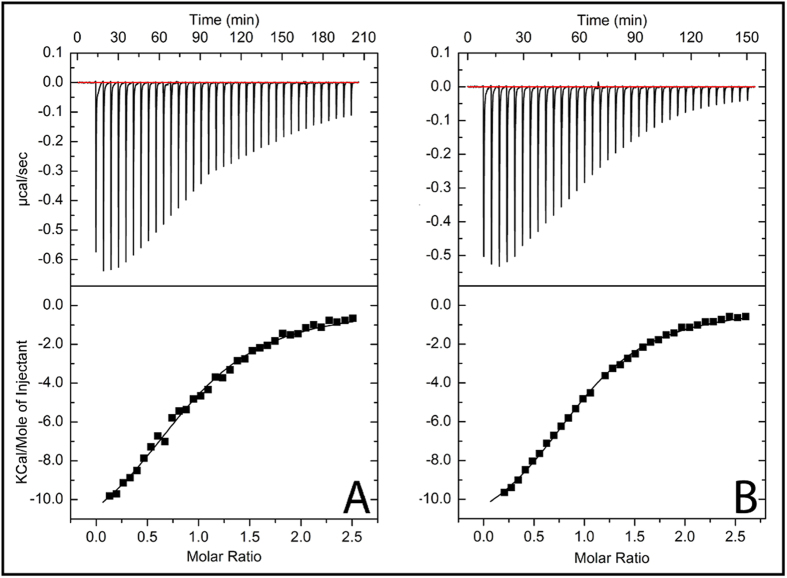
Thermodynamics of mutant protein heterocomplex formation. ITC data of (**A**) WD4_C1A2_ titrated with wild type Cu^+^-Atox1 and (**B**) Atox1_C1A2_ titrated with wild type Cu^+^-WD4. Thermodynamic parameters obtained from fitting of the binding isotherms in **A** and **B** are reported in Table 1.

To define the thermodynamics of formation of the two identified protein heterocomplexes Atox1_C1C2_-Cu-WD4_C1A2_ and Atox1_C1A2_-Cu-WD4_C1C2_ we turned to isothermal titration calorimetry, ITC. The analysis is simpler here than in previous ITC experiments^26^ since with these mutants we probe simple binding equilibria without the possibility of dissociation into products. Cu^+^-loaded wild type proteins in the syringe were titrated into the apo forms of the mutated partner protein in the cell (Fig. 3). Deconvoluted thermodynamic parameters (ΔG, ΔH and ΔS) for formation of the two mutant protein heterocomplexes are reported in Table 1. In support of the SEC data reporting on pseudo-equilibrium conditions, the K_D_ values determined from ITC and the apparent K_D_ values from SEC are in rough agreement. The observed discrepancy for one of the protein heterocomplexes highlights the problem of how to best analyze the SEC data (*e.g.,* peak maximum or integrate) to estimate absolute affinity values. Nonetheless, the SEC approach is well justified for comparative purposes when values to be compared are derived the same way.

## Discussion

Many processes in living systems (for example, metal homoeostasis) occur through transient, often weak, interactions among proteins. In these cases, the interaction is often associated with a small, negative change in free energy. If the free energy change was large and negative, the complex would be stable and non-transient, whereas if it was positive no interaction would occur. In particular, cellular Cu^+^ trafficking occurs through Cu^+^-mediated protein-protein interactions where Cu^+^ is transiently bound to sulfur ligands of Cys residues from both proteins of the heterocomplex. The free energy of formation of Cu^+^-mediated protein-protein complexes is the outcome of the balance of the metal-donor(s) bond energies, of the hydrophobic and hydrophilic interaction energies at the interface, and of the entropic implications of these interactions. Additional factors to consider are solvent effects, both at the metal site and the protein-protein interface, the deprotonation of sulfur ligands, and intra-protein structural rearrangements. To avoid protein-protein interactions in absence of metal, the contribution to the change in free energy resulting from protein-protein interactions alone must be positive. Thus, the determining energetic contribution leading to formation of detectable amounts of protein heterocomplex in the presence of Cu^+^ results from the involvement of amino acid side chains (from both proteins) in the coordination sphere of Cu^+^.

We recently determined the enthalpic and entropic contributions of the two steps in Fig. 1 that describes Cu^+^ transfer from wild type Atox1 to WD4^26^. The ensemble of heterocomplexes was there treated as one single species although it likely contains an equilibrated mixture of different Cu^+^-Cys coordinated protein heterocomplexes. Here we extend to the mechanistic level by determining exactly what Cu-Cys coordinated species are populated in the wild type Atox1-Cu-WD4 ensemble and by providing the first thermodynamic description of a Cu transfer reaction on a molecular level (see below).

The finding that only two sets of Cu^+^-Cys_3_ combinations govern protein heterocomplexes designates that the wild type protein heterocomplex ensemble will contain a mixture of these. The importance of C_1_ for the Cu^+^-mediated protein heterocomplexes between the yeast proteins Atx1 and the first Cu^+^-binding domain of the Ccc2 ATPase^27^ and, between human Atox1 and MK1 (the first Cu^+^-binding domain of Menke’s disease protein)^21^, has been reported previously using NMR experiments probing protein heterocomplex via increased reorientation time of molecular tumbling. We here show that the same principle is true in the Atox1-WD4 system. This is not necessarily expected since many features of homologous Cu^+^ transport proteins in different organisms have been found to vary. In our earlier QM-MM computations of Cu^+^ transfer from Atox1 to WD4 we assessed all possible bi-, tri-, and tetracoordinated Cu^+^-Cys protein heterocomplexes using restrained energy minimizations along given reaction coordinates^22^. The *in silico* work predicted that the most favorable pathway involved two tricoordinate protein heterocomplexes in which Cu^+^ was coordinated by Atox1_C1C2_ and WD4_C1_ in one and by Atox1_C1_ and WD4_C1C2_ in the other. These are the exact same tricoordinate species as those we observed here; thus, the QM-MM prediction and the *in vitro* experimental data are in outstanding agreement.

**Figure 4:**
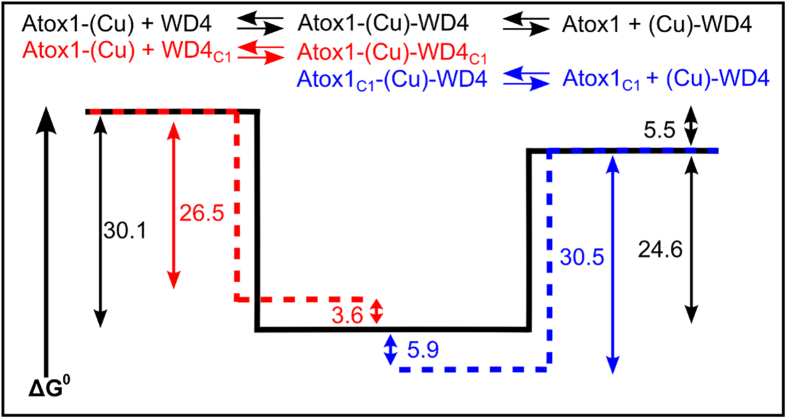
Free energy diagram for Cu^+^ transfer reaction in Figure 1. The reaction between Cu^+^-Atox1 and WD4_C1A2_ probes Step 1 (red dashed line), while the reaction of Cu^+^-WD4 and Atox1_C1A2_ probes the reverse of Step 2 (blue dashed line). The free energy values for Steps 1 and 2 of the wild type reaction are taken from^26^ (black line); the values for the mutant reactions are from Table 1. The free energy of the reactants (Cu^+^-Atox1 and WD4) as well as of the products (Atox1 and Cu^+^-WD4) in Fig. 1 are assumed to be the same in wild type and mutant reactions (reasonable since the Cu^+^ in these two samples is bound to the wild type protein). The comparison of free energy values shows that the Atox1_C1C2_-Cu-WD4_C1A2_ complex is higher, and the Atox1_C1A2_-Cu-WD4_C1C2_ complex is lower, in free energy as compared to the wild type Atox1-Cu-WD4 ensemble.

In Fig. 4, we have combined the apparent free energy values determined via SEC for formation of the two mutant heterocomplexes Atox1_C1C2_-Cu-WD4_C1A2_ and Atox1_C1A2_-Cu-WD4_C1C2_ with the same data for the wild type reaction in a diagram. Whereas formation of Atox1_C1C2_-Cu-WD4_C1A2_ reports on Step 1, formation of Atox1_C1A2_-Cu-WD4_C1C2_ reports on the reverse of Step 2 in Fig. 1. If we assume that the reactants and products are energetically the same in mutant and wild type mixtures (which is reasonable since the Cu^+^ is bound to the protein with an intact C_1_XXC_2_ site), it is clear that the Atox1_C1C2_-Cu-WD4_C1A2_ complex is higher in free energy and the Atox1_C1A2_-Cu-WD4_C1C2_ is lower in free energy than the wild type protein heterocomplex ensemble. If the latter is a mixture of Atox1_C1C2_-Cu-WD4_C1_ and Atox1_C1_-Cu-WD4_C1C2_ coordinated species, we can estimate from the free energy values that 60% of the heterocomplexes are Atox1_C1C2_-Cu-WD4_C1_ and 40% are Atox1_C1_-Cu-WD4_C1C2_.

**Figure 5:**
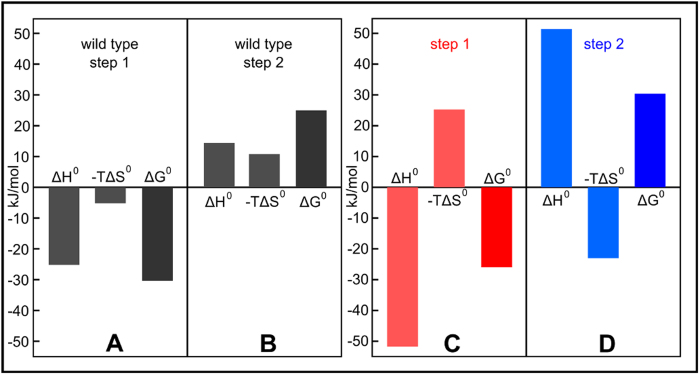
Enthalpic and entropic contributions to free energy changes of *Scheme 1*. Thermodynamics (ΔG, ΔH, ΔS) for Step 1 (reactants to heterocomplex, **A**) and Step 2 (heterocomplex to products, **B**) of the wild type Atox1-WD4 reaction taken from^26^. Thermodynamics (ΔG, ΔH, ΔS) for formation of Atox1_C1C2_-Cu-WD4_C1_ from the reactants in Fig. 1 (Cu-Atox1_C1C2_ + WD4_C1_) (step 1, red, **C**) and the inverse of the thermodynamics for formation of Atox1_C1_-Cu-WD4_C1C2_ from the products in Fig. 1 (Atox1_C1_ + Cu-WD4_C1C2_) (step 2, blue, **D**).

From the ITC data we revealed how the free energy change of complex formation is divided between entropy and enthalpy for the two mutant protein heterocomplexes. Again with the WD4_C1A2_ variant we assess Step 1 and with the Atox1_C1A2_ variant, we assess the reverse of Step 2 in Fig. 1. It emerges that both Step 1 and the reverse of Step 2 are driven by a large favorable enthalpies that are in part counteracted by unfavorable entropies (Fig. 5C,D). In contrast, for the wild type reaction both Step 1 and the reverse of Step 2 are driven by a combination of favorable enthalpy and entropy (Fig. 5A,B). Thus, instead of large opposing forces, the wild type system involves forces of lesser magnitudes. We speculate that this reduction in both favorable enthalpy and unfavorable entropy is due to rapid and continuous interconversion of the two Cu^+^ coordinations within the equilibrated wild type protein heterocomplex ensemble. Such dynamic interconversion within the protein heterocomplex ensemble will reduce the favorable enthalpy while the entropy increases. Thus although the free energy change associated with each step in Fig. 1 remains roughly the same, there is strong compensatory forces between enthalpy and entropy. The mechanistic explanation for this likely involves strength of the transient Cu^+^-Cys bonds, conformational dynamics of the system and in the Cu^+^ coordination sphere, and coupling with water/proton release/uptake at residues in the Cu^+^ site^28^. Further support for the presence of interconverting Cu^+^ ligands, and thus dynamics in the Cu^+^ coordination sphere, in the wt ensemble of protein heterocomplex is the distinct difference in near-UV CD signal of the wt protein heterocomplex ensemble as compared to the signals of the two mutant (with static Cu^+^ coordination) protein heterocomplexes (Fig. S3).

Enthalpy-entropy compensation is a common phenomenon in protein-ligand interactions that has been justified by shared features of the physical reaction mechanism and/or by an evolutionary advantage of free energy ‘buffering’^29^,^30^. Nonetheless, concern has been raised that some entropy-enthalpy compensation reports truly originated in experimental errors as the inaccuracy in ΔH may be up to 20% in normal ITC experiments^31^. It is therefore important to point out that our ΔH values differ by a factor of two, which is much more than 20%. In analogy to our finding, when Cu^2+^-ligating His residues were exchanged for Ala in the Aβ peptide, the observed enthalpy-entropy compensation for Cu^2+^ interactions with these Aβ variants was explained by coordination plasticity and redistribution of species^32^. In addition to protein-ligand interactions, our findings highlight the role of enthalpy-entropy compensation also in weak, transient protein-protein interactions.

In conclusion, we demonstrate that only two tricoordinate Atox1-Cu-WD4 complexes form *in vitro*, emphasizing the importance of C_1_ in both proteins for successful Cu^+^ transfer. Thermodynamic analysis discloses that enthalpy-entropy compensation is at play in the wild type protein heterocomplex ensemble. This is compatible with a dynamic interconversion of the two identified tricoordinate Cu^+^ sites which, in turn, supports the proposed Cu^+^ transfer mechanism^19^ involving rapid associative/dissociative exchange. Rapid and continuous switching between different Cu^+^ coordinations may be a way to shield against attack by unwanted Cu^+^ ligands in the solution (i.e., glutathione *in vivo*). Also, it may be easier to regulate the exact timing of the Cu^+^ transfer event to the target (here, WD4) if the heterocomplex ensemble is dynamic and not stuck in an enthalpic trap. Enthalpy-entropy compensation may be a common (yet unexplored) concept in transient protein-protein interactions that mediate cellular signals. Comparative studies with the other five metal-binding domains in WD are required before drawing general conclusions.

## Materials and Methods

### Protein production

Wild type forms of WD4 and Atox1 proteins were expressed and purified as reported previously^26^. The four mutants in which metal-binding site cysteines were changed one by one to alanines in both proteins (i.e., Atox1_C1A2_, Atox1_A1C2_, WD4_C1A2_, and WD4_A1C2_) were created by site-directed mutagenesis (GenScript USA Inc.), followed by purification via the same method as the corresponding wild type protein. In short, the centrifuged lysate was loaded onto 5 ml HP ion exchange columns (GE Healthcare): SP for Atox1 and Q for WD4. The corresponding elution peaks were further purified over a S30 preparative size-exclusion chromatography (SEC) column. All purification steps were performed in presence of 2 mM DTT and proteins eluted in their apo forms. Chromatography was performed on an ÄKTA purifier (GE Healthcare). The desired protein peak was concentrated, shock-frozen using liquid N_2_ and stored at −80 °C before use. Protein purity was confirmed by single bands on SDS-PAGE and single elution peaks on SEC. Protein concentrations were determined using ε_280_ of 1,550 and 2,980 M^−1^ cm^−1^ (based on amino acid sequence) for WD4 and Atox1 variants, respectively.

For holoprotein experiments (i.e., 1-to-1 Cu^+^-protein complexes), both Atox1 and WD4 were loaded with stoichiometric amounts of Cu^+^ (from a water stock of CuCl_2_) in presence of a 5-fold excess of DTT. This fulfills two purposes: to keep the Cys side chains in the proteins reduced and to reduce the Cu ions in solution from Cu^2+^ to Cu^+^, which, upon addition of protein, allows Cu^+^ to bind to the proteins. When Cu^+^ is loaded in the proteins, the resulting reduced holo forms are stable in buffer at aerobic conditions over the course of several hours to a day. The selected excess concentration of the reducing agent DTT (5–7 fold over protein) has been tested out previously to be high enough to keep the Cu^+^ ions reduced prior to protein binding but still low enough to allow for stoichiometric Cu^+^ loading of the proteins^26^,^33^. Stoichiometric 1:1 binding of Cu to wild type forms of Atox1 and WD4 were confirmed by near-UV CD and absorption titrations as in^26^. Although the selected buffer (Tris pH 7.6) may complex Cu^+^ ions, we have found this to be very slow (days) and not interfere with Cu^+^ loading of the proteins. After Cu^+^ incorporation in the proteins, the subsequent interactions between pairs of proteins do not involve Cu^+^ dissociation and, thus, the presence of Tris does not influence the estimated equilibrium constants.

### SEC

Similar to previous experiments on the wild type proteins^26^, analytical SEC experiments were performed with a Superdex 75 10/300 analytical column (volume of 24 ml) on an ÄKTA purifier (GE Healthcare) at 10 °C. The column was pre-equilibrated with 40 mM TrisHCl, 50 mM NaCl at pH 7.6. The protein samples were prepared in the same buffer but with the addition of 2 mM DTT. Sample was injected with a Hamilton SYN50018P syringe and a 100 μl injection loop. The elution profiles of protein samples were monitored using dual-channel absorption detection at 254 nm and 280 nm. A range of experiments was performed (as described in the text), such as mixture of apoproteins, individual holo-proteins, and Cu^+^ transfer mixtures. Experimental protein concentrations (of each protein) in the samples prior to SEC analysis ranged from 100 to 260 μM. Atox1 (apo and holo) elutes at 14.2 ml, WD4 (apo and holo) elutes at 12.9 ml and the protein heterocomplex elutes at 12.3 ml at our conditions and column. K_D_ values were calculated using the protein concentrations determined from 280 nm elution peaks and the 254/280 nm ratios (fraction apo versus holo) (see Results for detailed procedure).

### Data analysis

Experimental elution profiles were deconvoluted using the fityk software^34^. Individual protein peaks were fit using Gaussian peaks. Elution volumes for the individual proteins were fixed based on data from individual protein runs, while peak height and width floating. The protein heterocomplex was fit using a logarithmically skewed Gaussian with all parameters floating.

### ITC

ITC experiments were performed with an ITC_200_ (MicroCal) to probe the interaction between Atox1_C1A2_ and WT Cu-loaded WD4 and between WT Cu-loaded Atox1 and WD4_C1A2_. In a typical run, 35 automated injections of 1.11 μl syringe protein with 300s breaks in between injections were made at 25 °C and 600 rpm stirring speed in low feedback mode. Concentration of holoprotein (i.e., 1:1 mixing of Cu^+^ and wild type protein) in the syringe was 1.0–1.2 mM whereas the apoprotein (mutant protein) concentration in the cell was 80–85 μM. The buffer in both cell and syringe samples was 40 mM TrisHCl, 50 mM NaCl at pH 7.6 with 1 mM DTT. Data integration, fitting and evaluation were performed using the software Origin™ 7 (ITC_200_ plugin from MicroCal).

## Additional Information

**How to cite this article**: Niemiec, M. S. *et al*. Enthalpy-entropy compensation at play in human copper ion transfer. *Sci. Rep.* **5**, 10518; doi: 10.1038/srep10518 (2015).

## Supplementary Material

Supplementary Information

## Figures and Tables

**Table 1:** **Thermodynamic parameters for Atox1-WD4 Cu^+^ transfer.** ****

**Reaction mixture**	**Parameter**	**Step 1**	**Step 2**	**Overall**
Cu-Atox1_C1C2_ + WD4_C1C2_	ΔG_SEC_ (kJ/mol)	−30.1	+24.6	*−5.5*
	K_SEC_	0.42·10^6^ M^−1^	26.1·10^−6^ M	*11.0*
	ΔH_ITC_ (kJ/mol)	−25.1	+14.0	*−11.1*
	TΔS_ITC_ (kJ/mol)	+5.0	−10.6	*−5.6*
Cu-Atox1_C1C2_ + WD4_C1A2_	ΔG_SEC_ (kJ/mol)	−26.5		
	K_SEC_	0.09·10^6^ M^−1^		
	K_ITC_	0.05·10^6^ M^−1^		
	ΔH_ITC_ (kJ/mol)	−52		
	TΔS_ITC_ (kJ/mol)	−26		
Atox1_C1A2_ + Cu-WD4_C1C2_	ΔG_SEC_ (kJ/mol)		+30.5	
	K_SEC_		2·10^−6^ M	
	K_ITC_		17·10^−6^ M	
	ΔH_ITC_ (kJ/mol)		+52	
	TΔS_ITC_ (kJ/mol)		+24	

Step 1 and Step 2 relate to Fig. 1. The data for the wild type reaction, involving both Steps 1 and 2, is taken from^26^. The mutant data comes from SEC (Fig. 2; apparent K values estimated from concentrations derived from elution profiles) and ITC (Fig. 3; K, ΔH and ΔS was determined by fitting of ITC data to a 1:1 binding model) experiments and formation of Atox1_C1C2_-Cu-WD4_C1A2_ corresponds to Step 1 whereas dissociation of the Atox1_C1A2_-Cu-WD4_C1C2_ complex corresponds to Step 2 in Fig. 1. Errors may reach 5%. See text for further details.
