# Supplementary material for: Enthalpy-entropy compensation at play in human copper ion transfer
Source: Sci Rep. 2015 May 27;5:10518. doi: 10.1038/srep10518 (PMC4444973; doi:10.1038/srep10518)
Supplement: Supplementary Information [file srep10518-s1.pdf]

Supplemental Information

Enthalpy-entropy compensation at play in human copper ion transfer

Moritz S Niemiec, Artur PG Dingeldein, and Pernilla Wittung-Stafshede

Content:

Figure S1

Figure S2

Figure S3

Figure S1.

SEC analysis probed by dual wavelength absorption of Cu binding and Cu-dependent interaction of wild-type forms of Atox1 and WD4. While the absorbance at 280 nm remains constant upon Cu binding, the absorption at 254 nm increases. (**A**) and (**B**): Atox1 apo and holo, (**C**) and (**D**): WD4 apo and holo. (**E**)**:** Mixture of apo-Atox1 and apo-WD4; (**F**)**:** mixture of Cu-Atox1 and apo-WD4 (transfer mixture). (**G**)**:** Overlay of 280 nm absorbance elution profile of apo mixture (black) and transfer mixture (red). (**H**)**:** The SEC trace at 280 nm for the transfer mixture deconvoluted using fityk®: Experimental trace (black; named 'data'), as well as deconvoluted peaks for hetero-complex (blue), individual WD4 (red), individual Atox1 (green), and the sum of the three deconvoluted signals (grey). This figure is an illustration of published data[1].

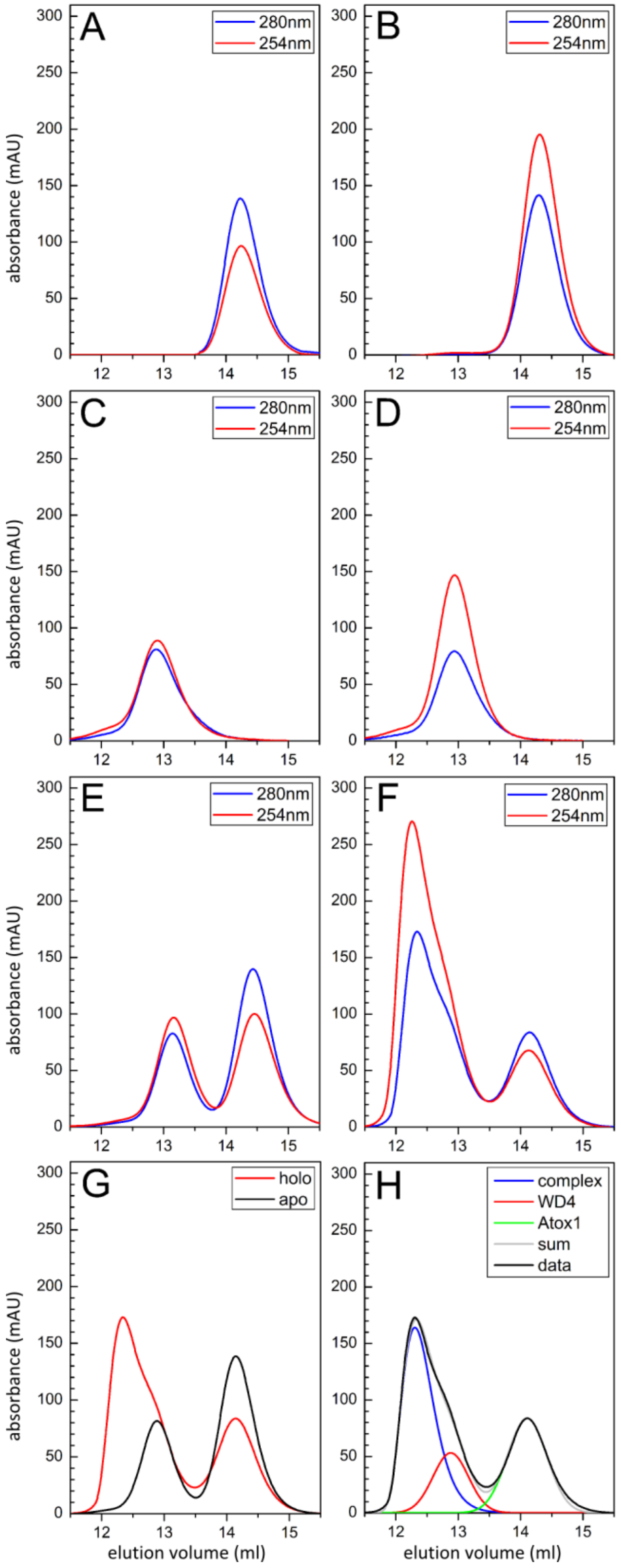

Figure S2.

Characterization of apo proteins. SEC traces (280 nm, blue; 254 nm, red) for apo mixtures of (A) wild-type Atox1 and $WD4_{C1A2}$, and (B) $Atox1_{C1A2}$ and wild-type WD4. The Atox1 proteins elute around 14.2 ml, whereas the WD4 proteins elute around 12.9 ml. No hetero-protein complexes are found in the absence of Cu (see **Figure 3CD** for elutions of these two mixtures when Cu is present). The data here also shows that the individual mutant proteins are folded, since they elute as single well defined peaks, in each case, at a position matching the corresponding wild-type protein. The same holds true for the $WD4_{A1C2}$ and $Atox1_{A1C2}$ variants, as demonstrated in **Figure 3AB**. Whereas wild-type WD4 has similar 254 and 280 nm absorbance values in its apo-form, Atox1 variants and the WD4 mutant have lower 254 nm absorbance as compared to the 280 nm absorbance. The 254 nm absorbance increases upon Cu binding to the wild-type proteins.

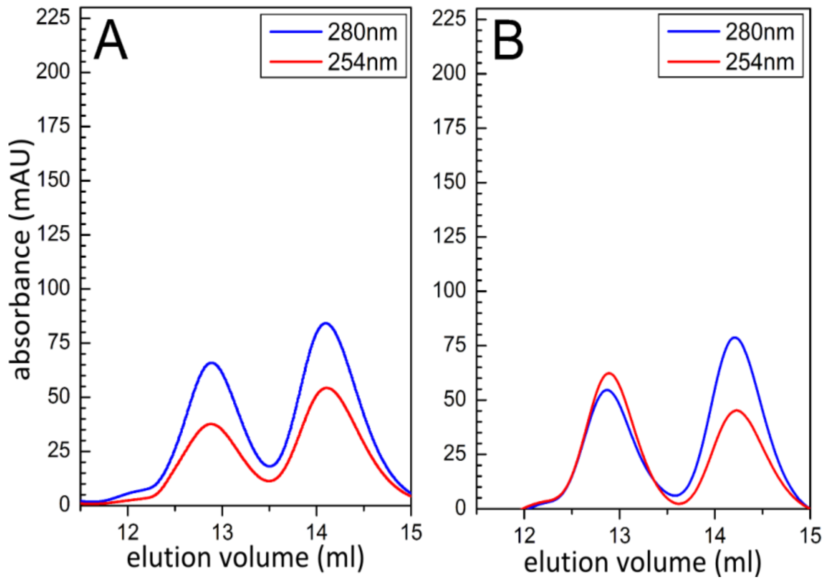

Figure S3.

Near UV CD signals of hetero-protein complexes scaled to the same concentration. Cu-Cys contributions appear in the 260-300 nm region. The isolated CD signal of each hetero-protein complex was defined by taking the CD of the mixture and subtracting the contributions from the monomeric apo/holo protein species (measured individually) in proportions determined via the known (from SEC) K_D values. As is evident from the data in the figure, the wild-type Atox1-Cu-WD4 hetero-protein complex signal differs from the other two signals such that they are almost mirror images. If the wild-type hetero-protein complex ensemble is a mixture of the two mutant hetero-complexes in terms of Cu-Cys coordinations, as proposed in this study, it is not a simple sum of two populations of stable Cu coordinations. Instead, a rapid inter-conversion of the two Cu coordinations within the wild-type hetero-protein complex ensemble can explain the difference in CD features since dynamic ligand switching may alter electronic/magnetic coupling around the Cu site.

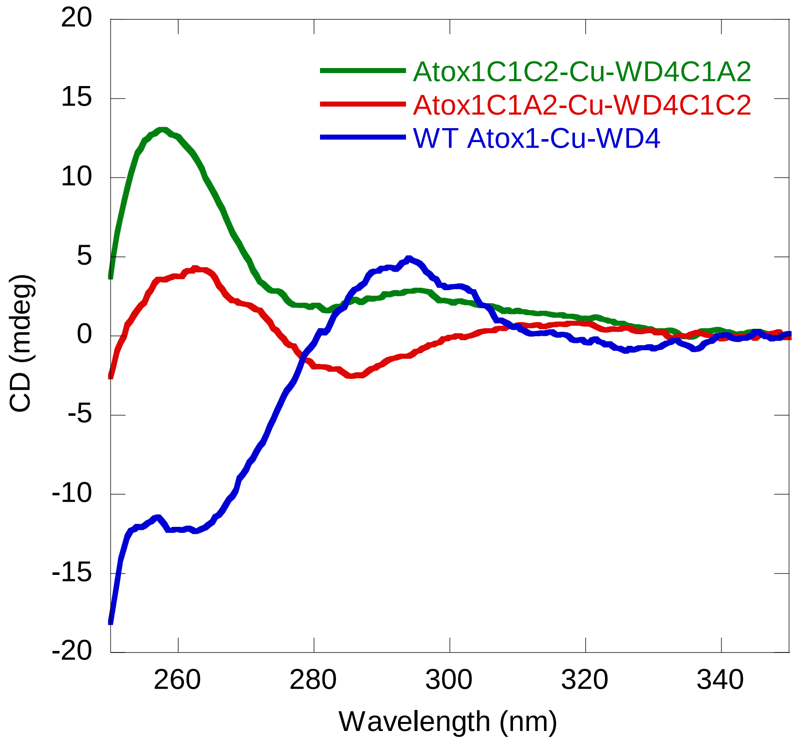

Reference

(1) Niemiec, M. S.; Weise, C. F.; Wittung-Stafshede, P. *PLoS One* **2012**, *7*, e36102.
